# Evanescent Field Controllable MZ Sensor via Femtosecond Laser Processing and Mechanic Polishing

**DOI:** 10.3390/mi12111421

**Published:** 2021-11-19

**Authors:** Zong-Da Zhang, Yan-Zhao Duan, Qi Guo, Si Gao, Bing-Rong Gao

**Affiliations:** State Key Laboratory of Integrated Optoelectronics, College of Electronic Science and Engineering, Jilin University, Changchun 130012, China; zhangzongda@jlu.edu.cn (Z.-D.Z.); duanyz20@mails.jlu.edu.cn (Y.-Z.D.); guoqi3351@163.com (Q.G.); gaosi18@mails.jlu.edu.cn (S.G.)

**Keywords:** femtosecond laser, waveguide, index sensor

## Abstract

Recently, optical sensors interacting with evanescent fields and the external environment around waveguides have attracted extensive attention. In the process of light propagation in the waveguide, the depth of the evanescent field is closely related to the accuracy of the optical sensor, and adjusting the depth of the evanescent field to obtain higher accuracy has become the primary challenge in fabricating on-chip optical sensors. In this study, the waveguide structure of a Mach–Zehnder interferometer was written directly in Corning Eagle 2000 borosilicate glass by a femtosecond laser, and the sensing window was exposed out of the bulk material by mechanical polishing. The refractive index detection device based on the proposed on-chip Mach–Zehnder interferometer has the advantages of small volume, light weight, and good stability. Its sensitivity can reach 206 nm/RIU or 337 dB/RIU, and the theoretical maximum measurement range is 1–1.508. Therefore, it can measure the refractive index quickly and accurately in extreme or complex environments, and has excellent application prospects.

## 1. Introduction

Optical sensing technology, a significant part of the sensor field, has been continuously improved and developed over the past few decades. Compared with traditional sensors, optical sensors have the advantages of reliable stability, high accuracy, and fast response. Optical sensors are widely used in aerospace [1], industrial and agricultural production [2,3,4,5], and medical devices [6,7,8,9,10], among other fields. On-chip optical sensors possess not only the advantages of ordinary optical sensors, but also the characteristics of small volume, light weight, and robustness against electromagnetic interference [11,12]. Therefore, devices such as the Mach–Zehnder (MZ) interferometer based on on-chip technology have good application prospects.

Mueller et al. fabricated Bragg gratings in optical fibers by electron beam exposure and selective etching, and combined them with microring resonators and Y-branch waveguides to fabricate a silicon photonic MZ sensor array for wavelength measurement [13]. Among them, the microring resonator plays the role of wavelength selection, and the Bragg grating reflects the light back to the input port to realize the equidistant wavelength distribution and sensing function. Khan et al. fabricated an MZ interferometer with a long-range surface plasmon waveguide structure in gold, and selectively etched a microfluidic channel to realize the sensing function by continuously changing the refractive index of the liquid in the channel [14].

**Figure 1:**
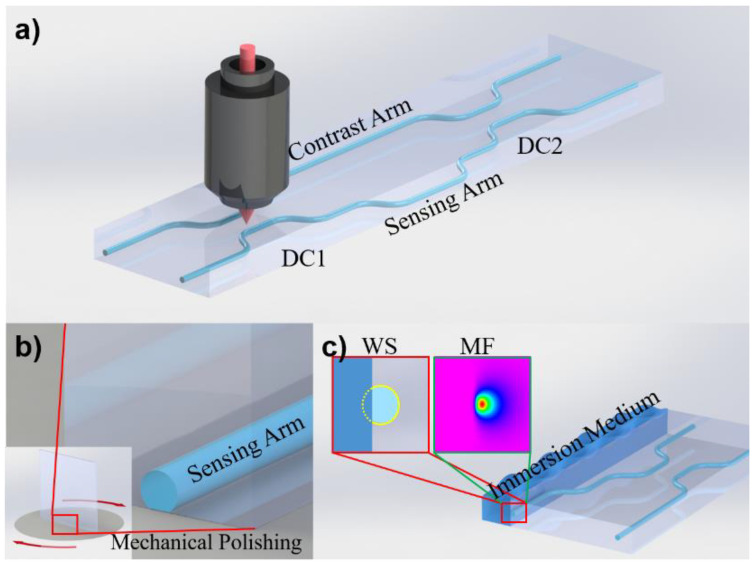
Schematic illustrations of (**a**) femtosecond laser direct writing of an asymmetric on-chip MZ interferometer, (**b**) mechanical polishing of the sensing arm side, and (**c**) the cross section of the device after immersion in the liquid to be measured. The front view of the waveguide section (WS) is shown in the red box, and the mode field (MF) distribution of the waveguide is shown in the green box.

Recently, femtosecond lasers have attracted extensive attention because of their high efficiency, high precision, and three-dimensional machining ability [15,16,17,18]. In this work, we used a femtosecond laser to write a waveguide structure in Corning Eagle 2000 glass at a repetition rate of 1 MHz and a speed of 40 mm/s [19]. Then, we fabricated an asymmetric Mach–Zehnder interferometer with two directional couplers (DCs), as shown in Figure 1a. We polished one side of the arm (the sensing arm) to expose it to the glass substrate. As shown with the yellow dotted line in Figure 1c, changing the polishing depth and immersing it in the external liquid can change the cladding structure of the waveguide, thus changing the effective refractive index and phase of light in it. Through coherent superposition with the other arm (the contrast arm), different interference phenomena are produced, and the light intensity of the latter-stage DC can be detected. The environmental refractive index can be tested according to different splitting ratios. In addition, we used a supercontinuum light source to test the structure and compared the sensitivity of different frequencies of light under the same external refractive index change.

## 2. Principles and Simulation

When light is incident on the interface of a medium, it is refracted and reflected. When total internal reflection occurs, the refraction wave still exists, and has the form of a traveling wave along the interface direction; however, in the direction perpendicular to the interface, the refraction wave decays sharply according to the exponential law, and is called an evanescent wave [20]. The length of the evanescent wave entering the second medium perpendicular to the interface is defined as the effective penetration depth *d* [21]:

(1) $$d=\frac{1}{\sqrt{{\left(\frac{\beta }{n_{1}}\right)}^{2}-{\left(\frac{2\pi n_{2}}{\lambda_{1}n_{1}}\right)}^{2}}}$$

where $\lambda_{1}$ is the wavelength of light propagating in the waveguide core, β is the waveguide transmission constant, $n_{21}=n_{2}/n_{1}$ is the ratio of the refractive index between the two media, $n_{1}$ is the refractive index of the waveguide core, and $n_{2}$ is the cladding refractive index.

Due to the different polishing depths, the cladding refractive index distributions of the sensing and contrast arms are also different, so the effective penetration depth, mode field size, effective refractive index, and other physical properties of the two arms are different.

The relationship between optical path and phase is

(2) $$\frac{N_{eff}L}{\lambda }=\frac{\varphi }{2\pi }$$

where $N_{eff}$ is the effective refractive index, and L is the propagation length. When the propagation length is constant, a change in the effective refractive index causes a change in phase. For the contrast arm, the transmitted light has a constant phase $\varphi_{0}$. Therefore, the phase difference between the two arms is $\Delta \varphi =\varphi_{0}-\varphi$.

The function of an optical element can be expressed as a matrix. For a 50/50 beam splitter, its function evenly distributes the light from any input port to two output ports, and adds a phase difference of π/2 when reflecting. At the same time, the matrix must follow the law of conservation of energy, so its function matrix should be a unitary matrix, that is, the product of its transposed conjugate matrix and itself is the identity matrix. The matrix is [22]

(3) $$\frac{1}{\sqrt{2}}\left[\begin{array}{cc} 1 & e^{i\frac{\pi }{2}}\\ e^{i\frac{\pi }{2}} & 1 \end{array}\right]=\frac{1}{\sqrt{2}}\left[\begin{array}{cc} 1 & i\\ i & 1 \end{array}\right]$$

The middle part of an MZ interferometer is considered a phase delay line because the only parameter of interest is the phase difference between the two arms [23]. We can set the phase change of the contrast arm as 0 and the phase change of the sensing arm as *φ*. The matrix is expressed as

(4) $$\left[\begin{array}{cc} 1 & 0\\ 0 & e^{i\varphi } \end{array}\right]$$

Therefore, the entire MZ interferometer can be expressed as a matrix:


(5) $$\frac{1}{\sqrt{2}}\left[\begin{array}{cc} 1 & i\\ i & 1 \end{array}\right]*\left[\begin{array}{cc} e^{-i\varphi } & 0\\ 0 & 1 \end{array}\right]*\frac{1}{\sqrt{2}}\left[\begin{array}{cc} 1 & i\\ i & 1 \end{array}\right]$$

When we input the light of [10] into the interferometer, the electric field intensity distribution at the end of the interferometer is

(6) $$\left[\begin{array}{c} \alpha \\ \beta \end{array}\right]=\frac{1}{2}\left[\begin{array}{c} e^{-i\varphi }-1\\ ie^{-i\varphi }+i \end{array}\right]$$

where *φ* = 2π*N_eff_L*/*λ*. From Equation (6), it can be inferred that the output light intensity varies with the effective refractive index.

**Figure 2:**
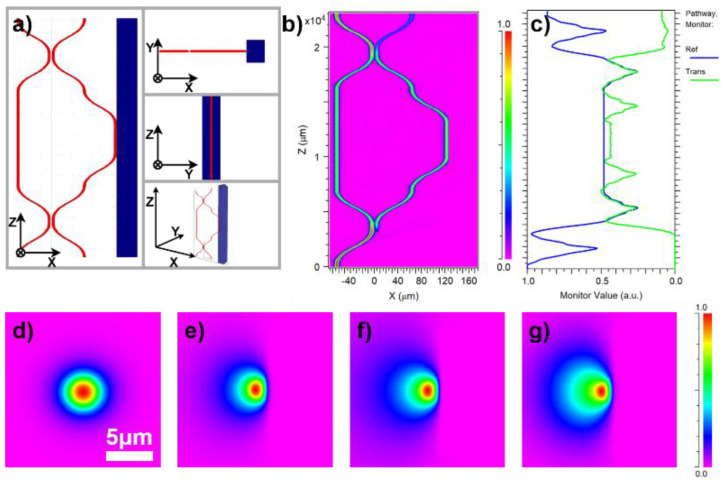
(**a**) Three views (x−z, y−z, x−y) and a 3D view of MZ interferometer in Rsoft CAD. (**b**) The energy distribution of 808 nm light propagating in an MZ interferometer. (**c**) The normalized energy evolution of the reference arm (blue line) and the sensing arm (green line). (**d**) The fundamental mode field distribution. (**e**,**f**) The mode field distribution in the sensing arm immersed in water and glycerol when the polishing depth is 33%. (**g**) The radiation mode in the sensing arm immersed in glycerol when the polishing depth is 45%.

We used Rsoft software to simulate the function of the proposed device [24,25,26,27]. The device structure was modeled as shown in Figure 2a, and the beam propagation method was used to simulate the light transmission in the sensor. In the simulation, the waveguide was assumed to be a refractive-index step waveguide. The total length of the structure is 25,000 μm, the grid size in the X and Y directions is 0.2 μm, and the grid size in the Z direction is 10 μm to reduce the calculation time. We use the default full transparent boundary condition (TBC) as the boundary condition of the calculation region. This condition allows the radiation wave to pass through the calculation boundary without reflection in order to prevent it from interfering with the simulation. First, the mode field distribution of the light in the contrast arm was calculated when the biased Gaussian light was input, and then the distribution was taken as the input light. At the same time, the external refractive index or polishing depth was continuously changed to detect the final splitting ratio.

The changes in the spectral ratio with the outside are shown in Figure 2c. It can be seen from the figure that the structure has high accuracy and high response speed for changes in the external refractive index. The longitudinal dimension of the device is 25 mm, and it takes only approximately 83 ps for light to pass through the sensor and change. We used the COMPUTE MODE module in Rsoft to calculate the transmission mode in the waveguide. The fundamental mode of the complete waveguide is shown in Figure 2d. For a polishing depth of 33% of the waveguide diameter (i.e., 1.65 μm), the internal mode field of the sensing arm after immersion in water and glycerol is shown in Figure 2e,f, respectively. With increasing polishing depth, the waveguide gradually loses its ability to limit light. For a polishing depth of 45% of the waveguide size (i.e., 2.25 μm), the mode field distribution in the sensing arm immersed in glycerol is shown in Figure 2g. It can be seen from the simulation that, before the waveguide loses its ability to limit light, a deeper polishing depth correlates with higher sensitivity.

## 3. Experiment and Results

**Figure 3:**
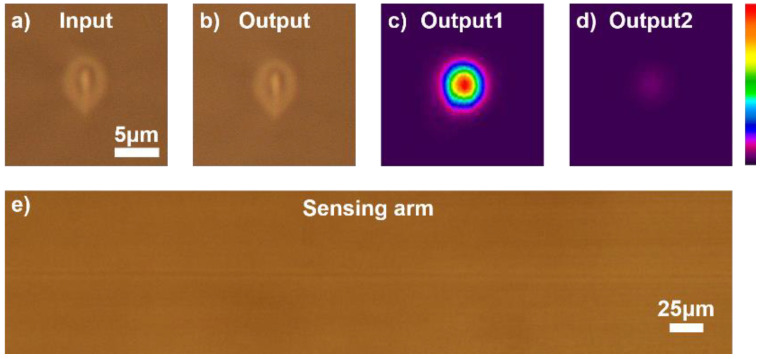
(**a**,**b**) Microscope photograph of femtosecond laser direct writing MZ interferometer input and output port. (**c**,**d**) Mode field photographs of the two output ports of the MZ interferometer without side polishing. (**e**) Microscope photograph of the sensing arm from the side view after side polishing.

The repetition frequency of the laser used in the experiment is 1 MHz [28], the pulse energy is 200 nJ, and the pulse width is 239 fs [29]. In order to eliminate the spherical aberration caused by refractive index mismatch to the greatest extent [30], we used an objective lens with spherical aberration compensation (Olympus, 40×, 0.75 N.A.). The waveguide was written inside Corning Eagle 2000 borosilicate glass (25 mm × 25 mm × 7 mm) at a depth of 0.17 mm. The relative position of the sample and the focus were controlled by an Aerotech A3200 motion control platform, and the MZ sensor structure was formed at a speed of 40 mm/s. To reduce the longitudinal coupling loss of the waveguide and remove the distortion of the waveguide structure caused by the poor quality of the focus near the edge of the sample, a mechanical polishing method was used to polish the waveguide section [31]. The cross-sectional photos of polished waveguide input and output ports are shown in Figure 3a,b, which indicate that the properties at both ends of the waveguide are exactly the same. The 808 nm wavelength light was coupled into the waveguide by a 10× objective lens with 0.25 N.A. and collected at the output end by the same objective lens. The mode field of the two output ports was measured using a single-mode analyzer (Spiricon BGP-USB-SP928-OSI) [32]. Mode field photographs are shown in Figure 3c,d. Before the sensing arm was polished, the ratio of the output light intensity at both ends was approximately 1:0, which means the phase difference between the two arms is 0.

The increase in the refractive index of the waveguide core is attributed to the thermal accumulation and thermal diffusion of the glass waveguide directly written by a femtosecond laser [33,34]. The diameter of the core layer is approximately the diameter of the focus, which can guide light because of the obvious increase in the refractive index due to the thermal accumulation of the focused femtosecond laser. The formation of the cladding structure is due to the joint action of thermal diffusion and thermal accumulation, which causes the material temperature outside the focus to exceed the deformation temperature of the glass, resulting in an increase in the refractive index. The temperature difference between the two positions makes the refractive indices of the two positions different. The position inside the focus has a higher refractive index, which acts as the core layer of the waveguide. The refractive index change outside the focus is smaller; therefore, it can limit light. The propagation loss of the waveguide is 0.3 dB/cm.

Next, the waveguide side was polished to reduce the size of the sensing arm in the X-direction, as shown in Figure 2b. As the entire waveguide structure was less than 190 µm on the surface of the glass substrate, when the waveguide was polished close to the side edge, the shadow generated by the glass edge under the microscope blocked the waveguide structure. Therefore, after fabrication of the waveguide structure, we used a femtosecond laser pulse to depict a group of equidistant and extremely fine-line arrays at the surface as the scale line of side polishing. This enabled realization of a polishing depth closer to the desired depth. A micrograph of the side-polished waveguide is shown in Figure 3e.

**Figure 4:**
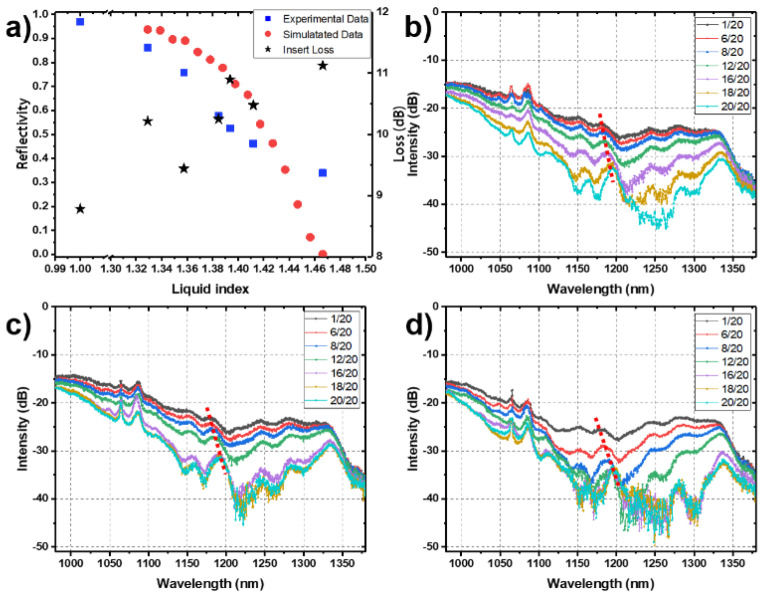
(**a**) Response of the device to 808 nm light. The blue squares represent the experimental test values, and the red dots represent the Rsoft simulation results. The black stars represent the device loss. (**b**–**d**) Spectral response of the device under different polishing depths (0.5 µm, 1 µm, and 1.5 µm, respectively); the lines of different colors represent the spectral response of the device to different refractive index matching solutions. (Refractive index matching liquid was made up of different proportions of water and glycerol; 1/20 means that the mixing ratio of water and glycerol was 1:19). The dotted line in the figure indicates a group of spectral peaks that move with the change in refractive index.

Then, we used the above-described test system to measure the device again, and used a power meter to detect the spectral ratio of the device. We used a mixture of ethanol and glycerol as the index-matching liquid to change the refractive index of the outside environment. Figure 4a shows the experimental and simulation results. The blue squares represent the experimental values, and the red dots represent the Rsoft simulation results, which agree closely with the experimental data. In addition, the black stars represent the insert loss, which increases with an increase in the external refractive index as the index difference between the core layer and the cladding layer decreases. In addition, the device is polarization sensitive because only the evanescent field in the X-direction of the sensing arm can penetrate into the refractive index liquid. When the TM light was injected, the light separation ratio of the TM light was nearly unchanged, whereas when the horizontal polarized light was introduced, the change in the ratio was the largest.

To further understand the response of the device to various wavelengths of electromagnetic waves, a UV-curable adhesive was used to bond the optical fibers at both ends of the device. To collect the output optical signal to the maximum extent, a 1550 nm single-mode fiber was bonded at the output port, because its diameter was slightly larger than the waveguide diameter of the device. To ensure better coupling strength at the input end, a 780 nm single-mode fiber was selected because the diameters of the two fibers were nearly the same, thereby minimizing the coupling loss. Finally, the fiber was connected to the supercontinuum light source by a fiber welding machine, and a spectrometer was connected at the output end to measure the response of each wavelength of light.

The experimental results are shown in Figure 4b–d. For the same polishing depth, a greater refractive index of the external liquid (glycerol accounts for a higher proportion) correlates with a greater device loss, which is caused by two factors. On the one hand, owing to the constant length of the sensing arm, the high refractive index of the external liquid leads to a larger optical path of the evanescent field when it propagates in the liquid. The optical path difference causes a change in phase, resulting in destructive interference when the output port is superimposed, resulting in a reduction in the light intensity of the optical outlet. On the other hand, a higher refractive index of the external liquid correlates with a weaker binding ability of the sensing arm to light, which leads to more light escaping from the sensing arm and a reduction in the light intensity at the light outlet. (The periodic small peak in the figure is caused by phase change, and its peak moves with the change in refractive index, as shown by the dotted line in the figure, whereas the overall downward trend is caused by loss.) At the same time, the separation degree of lines of different colors increases with increasing polishing depth. It can be seen from the figure that when the polishing depth is large and the refractive index of the external liquid is high, obvious noise appears. It is considered that the noise results from the slight unevenness of the sensing arm caused by mechanical polishing and the slight misalignment between the fiber and the waveguide. This indicates that the device has a small range when realizing precision measurement, while it has large range when the accuracy requirements of the measurement are low.

## 4. Conclusions

Using femtosecond laser direct writing waveguide technology, refractive index sensing devices based on an on-chip MZ interferometer can be fabricated rapidly and in batches. The mode field distribution of light propagating in the waveguide is related to the evanescent field of the signal light. When the refractive index of the surrounding environment of the interferometer changes, the evanescent field and effective refractive index of the signal light change, and the mode field distribution and propagation constant change, resulting in a change in the phase of the transmitted light. When coherent superposition occurs at the exit of the interferometer, the splitting ratio of the MZ interferometer is different. The structure has the advantages of small volume, light weight, and good stability. The sensitivity of the device is 207 nm/RIU or 337 dB/RIU. Although its sensitivity is lower than that of a optical fiber sensor, the borosilicate glass substrate ensures the strength and stability of the device, so that it can be used in harsh working environments, and can meet the needs of industrial production.
